# Parallel evolution of the G protein-coupled receptor GrlG and the loss of fruiting body formation in the social amoeba *Dictyostelium discoideum* evolved under low relatedness

**DOI:** 10.1093/g3journal/jkad235

**Published:** 2023-10-13

**Authors:** Laura M Walker, Rintsen N Sherpa, Sindhuri Ivaturi, Debra A Brock, Tyler J Larsen, Jason R Walker, Joan E Strassmann, David C Queller

**Affiliations:** Department of Biology, Washington University in St. Louis, St. Louis, MO 63130, USA; Department of Computational Medicine and Bioinformatics, University of Michigan, Ann Arbor, MI 48109, USA; Department of Biology, Washington University in St. Louis, St. Louis, MO 63130, USA; Department of Biology, Washington University in St. Louis, St. Louis, MO 63130, USA; Department of Biology, Washington University in St. Louis, St. Louis, MO 63130, USA; McDonnell Genome Institute, Washington University School of Medicine, St. Louis, MO 63130, USA; Department of Biology, Washington University in St. Louis, St. Louis, MO 63130, USA; Department of Biology, Washington University in St. Louis, St. Louis, MO 63130, USA

**Keywords:** aggregative multicellularity, *far2*, glutamate receptor-like protein, orphan GPCR

## Abstract

Aggregative multicellularity relies on cooperation among formerly independent cells to form a multicellular body. Previous work with *Dictyostelium discoideum* showed that experimental evolution under low relatedness profoundly decreased cooperation, as indicated by the loss of fruiting body formation in many clones and an increase of cheaters that contribute proportionally more to spores than to the dead stalk. Using whole-genome sequencing and variant analysis of these lines, we identified 38 single nucleotide polymorphisms in 29 genes. Each gene had 1 variant except for *grlG* (encoding a G protein-coupled receptor), which had 10 unique SNPs and 5 structural variants. Variants in the 5′ half of *grlG*—the region encoding the signal peptide and the extracellular binding domain—were significantly associated with the loss of fruiting body formation; the association was not significant in the 3′ half of the gene. These results suggest that the loss of *grlG* was adaptive under low relatedness and that at least the 5′ half of the gene is important for cooperation and multicellular development. This is surprising given some previous evidence that *grlG* encodes a folate receptor involved in predation, which occurs only during the single-celled stage. However, non-fruiting mutants showed little increase in a parallel evolution experiment where the multicellular stage was prevented from happening. This shows that non-fruiting mutants are not generally selected by any predation advantage but rather by something—likely cheating—during the multicellular stage.

## Introduction

High relatedness is crucial for the maintenance of cooperation in multicellularity. Without high relatedness to reduce conflict among individuals in a cooperative group, cheaters can evolve that exploit others and destabilize altruistic traits (Hardin 1968; Gilbert *et al*. 2007). High relatedness is easily achieved within organisms that go through a single-cell bottleneck or are otherwise clonal, as is true for most of the more than 20 transitions from unicellularity to multicellularity (Grosberg and Strathmann 2007; Knoll 2011). A single-cell origin thus reduces conflict, protects against cheaters, and promotes the division of labor that is so crucial for complex multicellularity such as is found in plants and animals (Grosberg and Strathmann 2007; Cooper and West 2018). However, a single-cell origin is not the only way to achieve multicellularity and its many benefits. Many other organisms from across the tree of life achieve multicellularity via aggregation or fusion of individual cells (Bonner 1998; Sebé-Pedrós *et al*. 2017). This form of multicellularity, called aggregative multicellularity, lacks a single-cell origin to ensure relatedness, so additional mechanisms are required to reduce conflict and maintain cooperation (Queller 2000).

Among the best studied organisms with aggregative multicellularity is the social amoeba *Dictyostelium discoideum* (Ostrowski 2019; Jahan *et al*. 2021). Though they are usually free-living in the soil, when faced with starvation, *D. discoideum* amoebae aggregate using the chemoattractant cAMP to cooperatively form a multicellular fruiting body in which about 80% develop into reproductive spores and the remaining 20% die forming a stalk (Kessin 2001). Stalk formation is altruistic because cells that contribute to the stalk die, sacrificing themselves to aid in lifting the spores, facilitating dispersal (smith *et al*. 2014). This partitioning of reproductive spores and dead stalk cells creates conflict in mixtures of genotypes by providing an opportunity for clones to cheat by contributing more than their fair share of cells to spores vs the stalk (Strassmann *et al*. 2000).

*Dictyostelium discoideum* is a particularly valuable and tractable model system for studying conflict (Strassmann and Queller 2011; Ostrowski 2019). This is due to an extensive set of experimental tools and established protocols (Eichinger and Rivero 2013; Fey *et al*. 2019) and the ability to manipulate intraorganismal relatedness by mixing genetically distinct amoebae. In the resulting multicellular fruiting bodies, often 1 clone (a cheater) contributes more to spores than stalk (Strassmann *et al*. 2000; Fortunato *et al*. 2003a; Buttery *et al*. 2009; Wolf *et al*. 2015; Madgwick *et al*. 2018).

Genetic mutations can also result in cheating (Santorelli *et al*. 2008). These mutations may pose a threat to cooperation and multicellular development, depending on the relatedness of aggregating cells and the extent to which those carrying the mutation can still cooperate on their own. Some cheaters are called “obligate” because they require another clone; they cannot form a fruiting body properly on their own. In a well-mixed (low-relatedness) population, these could increase to the point of losing cooperative fruiting (Buss 1982; Gilbert *et al*. 2007; Kuzdzal-Fick *et al*. 2011). If instead a clone can cheat when in chimera but cooperate when in isolation—a facultative cheater—then it can increase without threatening the collapse of multicellularity.

Selection under low relatedness increases the occurrence of both obligate (Ennis *et al*. 2000; Kuzdzal-Fick *et al*. 2011) and facultative cheaters (Santorelli *et al*. 2008). When obligate cheaters arise in the wild, where relatedness within aggregations is high (Gilbert *et al*. 2007), they should not persist because in nature they will usually find themselves in clonal or nearly clonal aggregations where their cheating advantage will not trump the disadvantage of their inability to form fruiting bodies on their own. In line with that expectation, no obligate cheaters have been identified from natural populations of *D. discoideum* (Gilbert *et al*. 2007). These findings are in accord with predictions of kin selection theory (Hamilton 1964) and show that high relatedness helps stabilize altruism in *D. discoideum*. The altruistic trait of stalk formation can be maintained only if the benefits are shared with related individuals who likely share the altruism allele.

Despite the great deal that has been learned about cooperation and conflict in *D. discoideum*, much remains to be learned with respect to the underlying genes and pathways. A number of genes involved in cheating phenotypes in *D. discoideum* have been found by screening knock-out mutant libraries generated by restriction enzyme-mediated integration (REMI) (Kuspa and Loomis 1992). The first cheater gene identified, *fbxA* (Ennis *et al*. 2000), remains as the only obligate cheater gene that has been characterized genetically. When cells from a clone carrying a mutant copy of *fbxA* (encoding F-box protein A) are mixed with wild-type cells, they become overrepresented as spores rather than stalk cells (Ennis *et al*. 2003). If allowed to spread, obligate cheater genes could destroy cooperation and even lead to population extinction (Fiegna and Velicer 2003). In addition, *fbxA* mutants are costly to the group as they carry a negative pleiotropic effect that decreases overall spore production as their frequency increases (Gilbert *et al*. 2007). When *fbxA* mutants are alone without others to exploit, no fruiting bodies are formed resulting in non-fruiting, obligate cheaters (Ennis *et al*. 2000, 2003). Non-fruiting, obligate cheaters like *fbxA* pose a great threat to multicellularity because fertile spore production requires a fruiting body.

In addition to the obligate cheater gene *fbxA*, numerous facultative cheater genes have been identified. Santorelli *et al*. (2008) screened a large REMI mutant library and identified over 100 genes predicted to cause facultative cheating when lost to mutation. Thus far, only 2 of the genes from that study have been characterized, *chtB* (Santorelli *et al*. 2013) and *chtC* (Khare and Shaulsky 2010). Mutant clones for both genes are able to cheat facultatively, increasing their numbers in chimera, but when alone, they contribute cells to both spores and stalk to form fruiting bodies normally and without any obvious fitness costs (Khare and Shaulsky 2010; Santorelli *et al*. 2013). However, 2 separately identified facultative cheater genes in *D. discoideum*, *dimA* and *csaA*, have disadvantageous pleiotropic effects that have been proposed to hinder their spread (Queller *et al*. 2003; Foster *et al*. 2004; Strassmann and Queller 2011).

Current data suggest that cheating can be accomplished in many ways. The cheater genes that have been identified thus far in *D. discoideum* share few sequence features or protein domains and are associated with a wide diversity of cellular functions and pathways (Santorelli *et al*. 2008). And although only a small number of those genes have been characterized, some mechanistic diversity is already apparent with the genes causing cheating via different means including altered communications in cell fate determination (Ennis *et al*. 2000; Thompson *et al*. 2004; Khare and Shaulsky 2010) and changes in cell adhesion (Queller *et al*. 2003). Perhaps that is to be expected given the complexity of multicellular development in *D. discoideum*. In addition to the aggregation and cooperation required among the previously free-living individuals, multicellular development involves the differentiation of cell types (*e.g.* prestalk and prespore), followed by maintenance of their appropriate proportions and coordination (Loomis 2015). Cheaters can presumably arise that exploit any number of aspects of this developmental process; the study of cheating therefore also has the potential to provide insight into development more broadly.

**Fig. 1.**
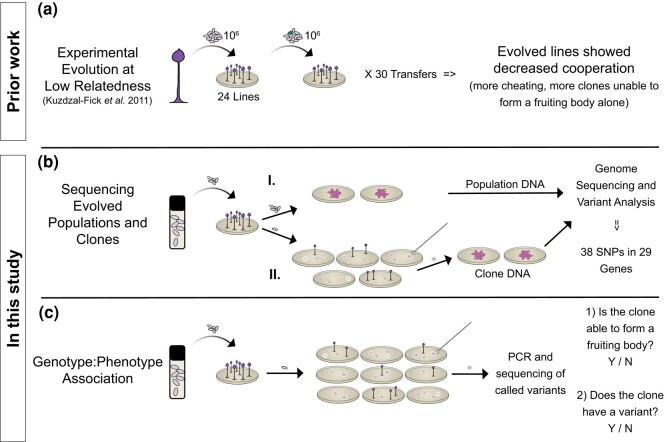
Outline of the experimental workflow. a) Experimental evolution of *D. discoideum* at low relatedness by Kuzdzal-Fick *et al*. (2011). A clonal isolate of AX4 was used to generate 24 replicate lines. After fruiting body formation, spores were collected, and 1 million thoroughly mixed spores were replated at each of the 31 passages (about 290 generations). Reestablishing low relatedness at each passage increased the likelihood that a new cheater mutation would be in close proximity to others lacking the mutation to exploit, thus allowing selection to favor mutations that conferred cheating. b) Whole-genome sequencing and variant analysis of the 24 evolved cell lines. For each evolved cell line, we sequenced (I.) the line in bulk, as a population that contains both fruiting and non-fruiting individuals, and (II.) a non-fruiting clone from each line. c) Association of variants with the loss of fruiting body formation in clones. We evaluated numerous clones from each line on 2 criteria: (1) whether or not they were able to form a fruiting body and (2) whether or not they carried the previously identified variant, via PCR and Sanger sequencing.

To expand our understanding of the genomics underlying cooperation and conflict during multicellular development in *D. discoideum*, in this study, we return to cell lines experimentally evolved under low relatedness by Kuzdzal-Fick *et al*. (2011). Starting from a single isolate of the wild-type lab strain AX4, Kuzdzal-Fick *et al*. (2011) established 24 replicate lines and grew them at low relatedness over 31 rounds (Fig. 1a). Each round began with 1 million spores which were allowed to hatch, proliferate by eating bacteria, and then form fruiting bodies. At each passage, the low relatedness was reestablished by replating a million thoroughly mixed spores. In this way, cheaters that appeared by mutation would not be with other such cells among the million and instead would more likely be in close proximity to cells that lack the mutation and can be exploited.

**Fig. 2.**
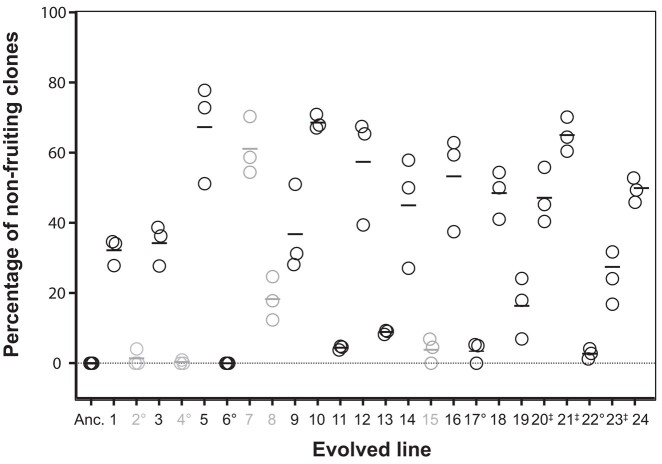
Percentage of non-fruiting clones per evolved cell line from Kuzdzal-Fick *et al*. (2011). After about 290 generations under low relatedness, 19 of the 24 lines had evolved to cheat their ancestor. The 5 lines (2, 4, 7, 8 and 18) that did not cheat are labeled with gray text). Non-fruiting, which was not present in the ancestor (Anc.), also increased in frequency to a varying degree among the evolved lines. The percentage of non-fruiting clones for each line (3 replicate measurements) is displayed in this scattered dot plot; the horizontal bar indicates the mean. In this study, we generated a whole-genome sequence for each evolved line in bulk as a population and for 1 non-fruiting clone from each, except where indicated (“°” indicates no clone was sequenced and “‡” indicates 2 clones sequenced). Modified from Kuzdzal-Fick *et al*. (2011).

The work by Kuzdzal-Fick *et al*. (2011) showed that drastically reduced relatedness allowed the spread of mutations that greatly decreased cooperation as indicated by the rise in cheating (seen in 19 of the 24 lines) and the concurrent rise of non-fruiting individuals (averaging 31% but rising as high as 69%; Fig. 2). Similar to the large group cost associated with *fbxA* mutants (Gilbert *et al*. 2007), spore production rapidly declined as the percentage of nonfruiters increased in chimeric mixtures with the ancestor (Kuzdzal-Fick *et al*. 2011). This demonstrates how low relatedness in a natural population could lead to a collapse of multicellularity and, with it, the advantages of fruiting body formation and spore dispersal.

In this study, we use whole-genome sequencing and variant analysis of these experimentally evolved *D. discoideum* cell lines to identify genomic changes that allowed decreased cooperation to evolve over the course of that experiment. In addition, we use a parallel evolution experiment to show that it is the selective advantage to cheat during multicellular development that likely drove the loss of fruiting body formation.

## Materials and methods

### Experimental cell lines

*Dictyostelium discoideum* cell lines were experimentally evolved under conditions of low relatedness by Kuzdzal-Fick *et al*. (2011) (Fig. 1a). Kuzdzal-Fick *et al*. (2011) froze spores from the evolved lines in KK2 buffer (2.25 g KH_2_HPO_4_ and 0.67 g K_2_HPO_4_ per L) with 25% glycerol and stored them at −80°. We thawed spores from the ancestor and from the final passage of each evolved line for genomic DNA extraction. Each evolved line is a population composed of cells and lineages carrying any newly acquired mutations. To capture all of this variation, we generated a bulk whole-genome sequence for each evolved line. We will refer to these sequences as populations. To narrow the focus to evolved variation most likely to be associated with the decreased cooperation in the evolved lines, we generated a whole-genome sequence for 1 non-fruiting clone from each evolved line (Fig. 1b). And finally, to identify new mutations that arose during the experimental evolution rather than standing variation, we generated a whole-genome sequence of the ancestor.

To isolate genomic DNA from each of the 24 evolved lines (Fig. 1b, I.), we plated spores onto 2 SM/5 agar plates [2 g glucose, 2 g BactoPeptone (Oxoid), 2 g yeast extract (Oxoid), 0.2 g MgCl_2_, 1.9 g KH_2_PO_4_, 1 g K_2_HPO_4_, and 15 g agar per liter] with 200 µL of *Klebsiella pneumoniae* in KK2 buffer (OD_600_ 1.5) as food. We incubated the plates at room temperature for ∼36 h or until log phase growth, before cells begin to aggregate. We then collected amoebae from the surface of 2 plates for each sample and washed them 4 to 5 times in chilled KK2 buffer to remove the food bacteria before DNA extraction.

To isolate genomic DNA from non-fruiting clones (Fig. 1b, II.), we first plated serial dilutions of spores to allow for clonal growth from individual spores. We inspected the plates daily to identify and mark all emerging clearings (plaques) resulting from germinating spores consuming local food bacteria and to ensure that plaques did not approach contacting one another. We aimed to sequence 1 non-fruiting clone from each evolved line but for 5 lines (2, 4, 6, 17, and 22); despite repeated attempts, we were unable to locate any non-fruiting clones (Fig. 2). To use the remaining sequencing space, we sequenced a second non-fruiting clone for 3 haphazardly selected lines (20, 21, and 23). After 3 to 5 days of growth, we collected cells from the leading edge of each non-fruiting clonal plaque using a sterile loop and plated them on SM/5 agar with *K. pneumoniae*. We then plated these cells to grow for DNA extraction using the same protocol described for the evolved lines. To document the non-fruiting morphology, we took photographs of each clonal plaque immediately before collecting cells from the leading edge for expansion (see Supplementary Fig. 2 for sample images). A second photograph was taken 1 to 2 days following collection, and we continued to monitor the plaques to ensure they never formed fruiting bodies.

### DNA isolation and sequencing

We isolated genomic DNA from the washed, log phase cells in the range of 1–2×10^8^ cells using the Qiagen DNeasy Blood and Tissue kit (Qiagen). We resuspended the genomic DNA in 10 mM Tris-HCl pH 8.5 and stored it at 4° until submission to the McDonnell Genome Institute at Washington University in St. Louis, MO, for library preparation and sequencing. Sequence libraries were prepared starting with 0.5 μg of genomic DNA using the KAPA Hyper Library Prep (KAPA Biosystems). We sequenced genomic DNA on the Illumina NovaSeq 6000 (150 bp × 2 paired-end) to an estimated depth of 100 × and 500 × for the clones and populations, respectively.

### Sequence alignment

We aligned the Illumina paired-end reads to a single FASTA file containing the reference genomes of both *D. discoideum* AX4 (GCF_000004695.1) and the food bacterium, *K. pneumoniae* (GCF_000240185.1_ASM24018v2) [both downloaded from the National Center for Biotechnology Information (NCBI) in June 2019]. We used BWA-MEM (0.7.15) (Li 2013) to index the *D. discoideum* and *K. pneumoniae*-concatenated reference genome and to align the paired-end reads for each sample.

We used an alignment pipeline to run BWA-MEM (Li and Durbin 2009) which converts, sorts, and indexes input, intermediate, and output formats using Picard v2.18.1 (http://broadinstitute.github.io/picard/), Sambamba v0.6.4 (Tarasov *et al*. 2015), and SAMtools v1.3.1 (using HTSLib v1.3.2) (Li *et al*. 2009) ultimately resulting in an aligned, sorted, compressed, and indexed CRAM file. We used both Picard (CollectInsertSizeMetrics, CollectAlignmentSummaryMetrics, CollectGcBiasmetrics) and SAMtools (flagstat) to evaluate alignment and coverage metrics using BAMs of the initial alignments including both *D. discoideum* and *K. pneumoniae* reference genome alignments and again after excluding reads that aligned to *K. pneumoniae*.

### Variant calling and filtration with GATK

We calculated the initial genotype likelihoods using GATK (4.1.2) (McKenna *et al*. 2010) HaplotypeCaller (-ERC GVCF --sample-ploidy 1) for each sample. Next, we ran GATK GenotypeGVCFs (--sample-ploidy 1) to create per-sample genotypes as individual VCF files for the entire *D. discoideum* and *K. pneumoniae*-concatenated reference genome. We selected the *D. discoideum* chromosomes (and the unplaced contigs associated with the reference genome) from the VCF files for downstream annotation and filtering using GATK SelectVariants. We used the Ensembl Variant Effect Predictor (VEP 95.3) (McLaren *et al*. 2016) to annotate all variants and add sequence ontology terms (--term SO) using the dicty2.7 assembly of “*Dictyostelium*_*discoideum*” from Ensembl Protists (release 43). We decomposed complex variants using vt decompose (Tan *et al*. 2015) before adding allele frequencies, merging, or filtering. We then processed the decomposed variants with bam-readcount (0.7.4) (https://github.com/genome/bam-readcount) and cyvcf2 (https://github.com/brentp/cyvcf2) to generate allele frequencies. We used VAtools (https://github.com/griffithlab/VAtools, 3.1.0) to add the AF (allele frequency) format fields to each sample including the allele frequencies of each allele as calculated by bam-readcount allele counts (ACs). We processed all per-sample VCFs with bgzip and tabix for speed and storage before merging to generate the full per-sample call set VCF using GATK (3.6) CombineVariants (-genotypeMergeOptions UNIQUIFY).

We performed variant filtration of the GATK VCF using bcftools v1.12 (using HTSLib v1.12) (Li 2011) and GATK. We did not consider indels or sites with more than one alternate allele. We are only interested in variation that arose during the course of experimental evolution or new variation between the ancestor and the evolved lines. To exclude preexisting variation, we first removed sites (from all samples) for which the ancestor was called as a variant (*i.e.* sites in the ancestor that differed from the reference genome) as well as sites that were left uncalled in the ancestor. To remove ancestral polymorphism, we next calculated the major allele frequency (MAF) of all sites in the ancestor BAM file using bam-readcount (0.7.4) (https://github.com/genome/bam-readcount). Using that information, we then removed sites (from all samples) for which, in the ancestor, the MAF < 0.90.

We applied hard filters using GATK VariantFiltration and SelectVariants following the GATK Best Practices standard recommendations (QD > 2, FS < 60, SOR < 3, MQ > 40, MQRankSum > −12.5 and ReadPosRankSum > −8). Next, we applied custom filters using bcftools view. The first custom filter was the removal of sites missing too much data, which we defined as sites left uncalled in more than 10 samples. For each of the 24 evolved lines, we sequenced the whole line (as a population), and for most of the lines, we also sequenced 1 non-fruiting clone, for a total of 2 samples per line (or a total of 3 samples for lines 20, 21, and 23 for which we sequenced 2 clones). There is a low likelihood that the same SNP will occur by chance in more than one evolved line. But because we sequenced 2 or 3 samples for each line (the population and individual clones), the maximum number of times that a SNP is likely to appear in our data is twice (or 3 times for lines 20, 21, and 23). For this reason, we applied a maximum alternate AC of 3. Next, we applied a minimum Phred-based quality score (QUAL) of 200 to remove low-quality sites and we removed sites with more than 1.5 times the average approximate read depth (DP) to reduce false positives. Last, we manually reviewed this final set of SNPs using the Integrative Genomics Viewer (IGV) to further reduce the number of false positives and misclassifications (Robinson *et al*. 2011, 2017).

### Variant calling and filtration with Freebayes

We also performed variant calling on all samples (joint calling) using Freebayes v1.3.1-dirty (Garrison and Marth 2012). We used the default parameters but for the following exceptions: sample ploidy of 1, pooled continuous mode, minimum base quality 10, and minimum mapping quality 10, and we only retained the best of 6 alleles. We streamed variant calls directly through the vcflib (Garrison *et al*. 2021) vcffilter, to remove variants with a quality score below 20. We decomposed complex variants into their constituent SNPs and indels using vcflib vcfallelicprimitives followed by normalization with vt (Tan *et al*. 2015).

We performed variant filtration of the normalized Freebayes VCF using bcftools v1.12 (using HTSLib v1.12) (Li 2011) following a similar process to that used for filtering the GATK VCF. Although the 2 callers calculate and output some different metrics for quality assessment, we generated filters resembling those applied to the GATK VCF as much as possible. First, we excluded indels and sites with more than one alternate allele, followed by the removal of background variation as described for the GATK VCF (ancestral sites with an MAF < 0.90 or sites in the ancestor that were either called as a variant or left uncalled). Next, we applied hard filters to remove calls affected by mapping quality or strand bias (MQM > 40, SAF > 0 and SAR > 0, SAP > 0.5 and SRP > 0.5, RPR > 1 and RPL > 1). We then applied the same set of custom filters to the Freebayes VCF as described for the GATK VCF including the removal of sites with more than 10 missing samples, sites with an alternate AC >3, and sites with a Phred-based quality score (QUAL) below 200. Finally, based on the approximate read depth (DP) in this remaining set of variants, we removed sites with more than 1.5 times the average to reduce false positives. To further reduce the number of false positives and misclassifications, we manually reviewed this final set of SNPs using the IGV (Robinson *et al*. 2011, 2017).

### The intersection of GATK and Freebayes VCFs and variant read support

We viewed the 2 separately generated and filtered VCF files (from GATK and Freebayes) side by side and removed 4 sites that were not present in both files. The SNPs we removed included 1 SNP that had only been initially called by one of the callers and 3 SNPs that, although initially called by both callers, only survived the filtration in one of the separate filtering pipelines. Going forward, we worked with these cross-validated SNPs in the annotated GATK-generated VCF.

As one final verification of variant support and quality, we confirmed that each variant that had been called in a non-fruiting clone could also be detected in its origin population (or the line from which the clone was isolated). To do this, we generated read counts for each SNP across all samples using bam-readcount v0.7.4 (https://github.com/genome/bam-readcount). We removed any SNP for which the origin population did not support the same alternate allele with at least 5 reads, which we expect would only occur for false positives or for SNPs that did not provide a selective advantage. Also, because we expect that most true variants will be unique to 1 replicate line, for each SNP, we viewed the number of reads supporting the same alternate allele across all samples. This led to the removal of 1 SNP that shared low level support for the same allele across multiple lines and also the rejection of 1 sample from a variant call (for details, see *Variant Read Support* in the Supplementary Supporting Information).

### Functional annotation clustering of genes with SNPs

We performed functional annotation enrichment analysis with the final set of 29 genes containing SNPs using the online tool, Database for Annotation, Visualization and Integrated Discovery (DAVID) (https://david.ncifcrf.gov/home.jsp; Huang *et al*. 2009a, 2009b). We determined enriched terms using a Benjamini-corrected *P*-value (< 0.05) (Benjamini and Hochberg 1995).

### Structural variant calling and filtration with Delly

We called structural variants (SVs) with Delly v0.8.3 (Rausch *et al*. 2012) following the Germline SV Calling workflow. We first did per-sample SV calling, providing the indexed, sorted, and duplicate-marked BAM files and the indexed *D. discoideum* and *K. pneumoniae-*concatenated reference genome. We then merged the SV sites to a single list and called genotypes across all samples. We merged all samples genotypes using bcftools v1.12 (using HTSLib v1.12) (Li 2011). We applied the Delly filter (-f germline) and then removed low-quality variants that were not flagged as “PASS” in the VCF filter field [PE > 3 (or PE > 5 for translocations) and QUAL ≥ 20]. We annotated the SVs using the Ensembl Variant Effect Predictor (VEP 95.3) as described for the GATK VCF. We only retained simple, intrachromosomal SVs. As described for the SNPs, the same SV is unlikely to occur in more than one evolved line. But because we sequenced 2 or 3 samples for each line (the population and individual clones), the maximum number of times that an SV is likely to appear in our data is twice (or 3 times for lines 20, 21, and 23). For this reason, we discarded SVs that were called in more than 3 samples. Last, as described for the SNPs, we manually reviewed this final set of SVs in IGV (Robinson *et al*. 2011).

### Association of variants with the loss of fruiting body formation

To investigate the potential correlation between called variants and the evolved decrease in cooperation represented by the inability to fruit, we returned to the evolved lines to isolate additional clones for genotyping (Fig. 1c). Each evolved line is a mixed population for both fruiters and nonfruiters (though the latter cannot be seen in mixtures), as well as for variants at a candidate locus. To test whether a mutation is associated with non-fruiting, we clonally plated the evolved lines carrying the called variants of interest and scored a total of 167 additional clones for (1) whether or not they were able to form a fruiting body in isolation and (2) the presence or absence of the called variant(s).

For this analysis, we plated the evolved cell lines clonally (as described in the Experimental cell lines section) to allow for growth from individual spores. After 3 to 5 days of growth, we screened clones via PCR and Sanger sequencing. We took photographs of the screened clones to document the presence or absence of fruiting body formation (example images are available in Supplementary Fig. 2).

We generated genomic DNA for PCR genotyping clones using 1 of 2 methods. For some of the clones, we first collected cells from the leading edge of the plaque, grew them to larger numbers, and carried out a formal DNA extraction with the DNeasy Blood and Tissue kit (Qiagen). To increase throughput, for most clones, we directly lysed cells from the leading edge of the plaque and used the lysate for what we called a “plaque PCR.” Based on a protocol described by Charette and Cosson (2004), the plaque PCR included 2 steps. First, we used a sterile pipette tip to collect a small number of cells from the leading edge of a plaque and placed it in a tube containing 20 μL lysis buffer [10 mM Tris, pH 8.3, 50 mM KCl, 2.5 mM MgCl_2_, 0.45% Nonidet P-40 (NP40), and 0.45% Tween 20] with PK (1 μL of 20 μg/μL of PK for every 25 μL of lysis buffer). Next, we incubated the cells in lysis buffer for 1 min at 95° to inactivate the PK after which we used the cell lysate for PCR or stored them at −20°.

We designed primers to PCR-amplify the region spanning each called variant of interest (Supplementary Table 1). For the PCR, we used either 1 μL of cell lysate or ∼10 ng of DNA for the formal DNA isolations and the following reaction components: MgCl_2_ (25 mM) 1 µL, dNTPs (10 mM each) 0.5 µL, 5 pM of each primer, 5 × GoTaq Flexi Buffer 5 µL, GoTaq DNA Polymerase 0.2 µL, and H_2_O 10.3 µL for a 20 µL reaction. We used the following PCR protocol (adjusting the annealing temperature as needed, according to primer pairing): 95°, 2:00; 95°, 0:15; 50°, 0:15; and 60°, 3:00; repeat steps 2–4 34×, 60°, 5:00; 4° hold. We submitted PCR products and primers (the same primers used for amplification) to Genewiz (South Plainfield, NJ) for purification and Sanger sequencing. We trimmed the returned sequences with 4Peaks (https://nucleobytes.com/4peaks/) for alignment using SeaView (http://doua.prabi.fr/software/seaview).

We prioritized screening of variants that we had identified in evolved lines with a moderate percentage of non-fruiting clones (Fig. 2) so that both fruiting and non-fruiting clones could be included. We also prioritized variants with moderate allele frequencies in their evolved populations. The number of clones screened from each line varies according to the availability of fruiting and non-fruiting clones. The full list of variants and clones screened are available in Supplementary Table 2. For each line included in this screen, we tallied the number of clones that did and did not carry the called mutation(s) and whether or not the clone was able to form a fruiting body when plated clonally. We tested for significance using Fisher's exact test with a 95% confidence interval using GraphPad Prism (version 9.3 for MacOS).

### Association of the loss of fruiting body formation with selection during the social cycle

To investigate the potential connection between the loss of fruiting body formation and selection for cheaters, we additionally looked for non-fruiting clones in *D. discoideum* lines from a forthcoming experimental evolution study that was similar to Kuzdzal-Fick *et al*. (2011) but omitted the fruiting body stage, eliminating selection for cheating. Wild *D. discoideum* strains were experimentally evolved at low relatedness and replated every 48 h (30 rounds) by collecting entire plate contents and diluting by a factor of 200. Like the lines from Kuzdzal-Fick *et al*. (2011) that are the primary focus of this study, these lines evolved under low relatedness due to being thoroughly mixed at each passage. However, the short 48-h interval between each passage prevented these lines from undergoing fruiting body formation and so eliminated any benefit that might be gained by cheating. Thus, if nonfruiters tend to be cheaters as previous studies have generally shown (Ennis *et al*. 2000; Kuzdzal-Fick *et al*. 2011), few of them should be selected under these conditions.

Culture conditions and materials (SM/5 agar plates, KK2 buffer, *K. pneumoniae* food bacteria) for this experiment are as described in the *Experimental cell lines* section, feyunless stated otherwise. Starting from clonal isolates from each of the wild *D. discoideum* strains QS6, QS9, and QS18 (Supplementary Table 3), 3 replicate experimentally evolved lines were generated.

To estimate the prevalence of non-fruiting clones within these evolved lines, we plated serial dilutions of spores on 10 plates from the 3 ancestors and each of their 3 experimentally evolved lines (as described for the clone isolations for the main experiment, in the Experimental cell lines section). After 5 days, we photographed each plate and calculated the fraction of clonal plaques with absent or conspicuously deformed fruiting bodies (see an example image in Supplementary Fig. 3). We did 3 replicates of this assay (on 3 different days) for a total of 120 plates per strain (with the exception of QS9, which had 90 plates due to difficulties reviving one of its evolved lines from the freezer) for a total of 958, 324, and 778 clones from strains QS6, QS9, and QS18, respectively.

### Predicted protein structure

To explore proteins of interest, we first downloaded the amino acid sequences from DictyBase (Fey *et al*. 2013) and submitted them to Protter (Omasits *et al*. 2014) for visualization of membrane topology. We inspected protein features using UniProt (https://www.uniprot.org/) (UniProt Consortium *et al*. 2023). We also assessed 3-dimensional structural predictions generated by AlphaFold (https://alphafold.ebi.ac.uk/) (Jumper *et al*. 2021; Varadi *et al*. 2022).

## Results

### Raw data generation and alignment

We obtained over 5 trillion reads across all samples, with a high rate of alignment (94.7%) to the concatenated *D. discoideum* and *K. pneumoniae* reference genomes. All reads that aligned to *K. pneumoniae* (food bacterium) were excluded from further analysis. The remaining 2.7 trillion reads aligned to *D. discoideum* with an average rate of 89.3%. Read alignment was, on average, equally successful for clones and evolved lines (89.3 and 89.4%, respectively). The average mapped read depth of all annotated genes normalized by gene length (gene annotations downloaded from NCBI on 2019 October 25; Supplementary File 4) is 54 × and 195 × for clones and evolved lines, respectively (Table 1). The average mapped read depth of normalized intergenic regions is very similar with averages of 53 × and 180 × for clones and evolved lines, respectively.

**Table 1.** Number of SNPs and average read depth by sample. Whole-genome sequencing and variant analysis resulted in 38 SNPs called in 25 of the 47 samples and distributed throughout 17 of the 24 evolved lines. This table indicates the number of SNPs called for each sample. The average mapped read depth was calculated for the raw reads normalized by the length of all annotated genes. Samples from each evolved line, sequenced as populations, are simply numbered 1–24. Clonal samples isolated from each of the evolved lines are named according to the population number hyphenated with the non-fruiting clone ID (*e.g.* “NF1”). The non-fruiting clone IDs were retained for record keeping purposes; they are not related to the number of clones sequenced for a line.

Sample	SNP count	Average mapped read depth
1	0	128
1-NF2	0	43
2	1	137
3	0	146
3-NF1	1	127
4	0	171
5	0	161
5-NF2	3	40
6	1	152
7	4	229
7-NF2	5	44
8	0	165
8-NF1	0	89
9	1	220
9-NF3	1	24
10	1	151
10-NF3	1	23
11	2	116
11-NF1	0	37
12	1	215
12-NF1	1	97
13	1	165
13-NF2	1	70
14	0	127
14-NF3	0	22
15	0	176
15-NF1	0	69
16	0	277
16-NF2	3	118
17	3	123
18	0	191
18-NF1	3	30
19	0	319
19-NF1	0	80
20	0	231
20-NF1	1	66
20-NF3	1	32
21	1	272
21-NF1	3	40
21-NF2	5	45
22	3	291
23	0	305
23-NF1	0	25
23-NF2	0	43
24	0	222
24-NF1	1	32

### Resulting SNPs are selection driven

We independently filtered the variants called by GATK and Freebayes (the number of raw and filtered variants is available in Supplementary Table 4) and manually reviewed all remaining SNPs in IGV resulting in ∼75 sites in each call set. Finally, we removed any sites that did not cooccur in both call sets and/or sites lacking read support (described in The intersection of GATK and Freebayes VCFs and variant read support section). The final set of SNPs contained 38 biallelic SNPs associated with 29 different genes (Table 2). Each gene in this list has only 1 SNP except for the gene, *grlG* (DDB_G0272244), which has 10 unique SNPs (discussed in detail in the following sections). All SNPs are unique to 1 evolved line with the exception of 1 in the unannotated gene, DDB_G0276529, which was called in 2 lines (7 and 16). Most evolved lines have between 1 and 3 SNPs except for lines 7 and 21, which have 5 and 8 SNPs each, respectively (Table 1).

**Table 2.** List of called SNPs and the impacted locus. Whole-genome sequencing and variant analysis resulted in this list of 38 biallelic SNPs impacting 29 different genes. For each SNP, this table provides the precise location in the genome and the Dictybase gene ID (followed by gene name when available), followed by the called samples. Samples from each evolved line, sequenced as populations, are simply numbered 1–24. Clonal samples isolated from each of the evolved lines are named according to the population number hyphenated with the non-fruiting clone ID (*e.g.* “NF1”). The next 2 columns of the table are the variant consequence (VEP Consequence) and the estimated impact rating (VEP Impact) made by the Ensembl Variant Effect Predictor (VEP) (VEP 95.3). The final column indicates the amino acid change resulting from each coding variant.

Chrom:Site	Gene ID	Sample(s)	VEP Consequence	VEP Impact	Amino Acid Change
NC_007087.3:2556092	DDB_G0269332	22	missense_var.	Moderate	D/N
NC_007087.3:3010110	DDB_G0270828	21-NF1	missense_var.	Moderate	L/F
NC_007087.3:3624901	DDB_G0270964	18-NF1	missense_var.	Moderate	N/H
NC_007087.3:3928085	DDB_G0269956 (RTE)	21-NF2	upstream_gene_var.	Modifier	—
NC_007087.3:4563335	DDB_G0270834 (*sgmA*)	5-NF2	missense_var.	Moderate	L/F
NC_007088.5:1741979	DDB_G0272244 (*grlG*)	6	stop_gained	High	L/*
NC_007088.5:1741982	DDB_G0272244 (*grlG*)	7 & 7-NF2	missense_var.	Moderate	P/R
NC_007088.5:1742043	DDB_G0272244 (*grlG*)	11	missense_var.	Moderate	E/K
NC_007088.5:1742355	DDB_G0272244 (*grlG*)	17	missense_var.	Moderate	I/F
NC_007088.5:1742362	DDB_G0272244 (*grlG*)	13 & 13-NF2	missense_var.	Moderate	N/K
NC_007088.5:1742375	DDB_G0272244 (*grlG*)	10 & 10-NF3	stop_gained	High	L/*
NC_007088.5:1742506	DDB_G0272244 (*grlG*)	18-NF1	missense_var.	Moderate	L/F
NC_007088.5:1743478	DDB_G0272244 (*grlG*)	2	missense_var.	Moderate	Y/N
NC_007088.5:1743823	DDB_G0272244 (*grlG*)	21 & 21-NF2	stop_gained	High	G/*
NC_007088.5:1743844	DDB_G0272244 (*grlG*)	22	stop_gained	High	K/*
NC_007088.5:1761939	DDB_G0272484	16-NF2	missense_var.	Moderate	E/Q
NC_007088.5:4313995	DDB_G0274875 (*rnf160*)	16-NF2	stop_gained	High	E/*
NC_007088.5:6565220	DDB_G0276291	17	missense_var.	Moderate	K/N
NC_007088.5:6762467	DDB_G0276367	17	stop_gained	High	W/*
NC_007088.5:6787007	DDB_G0276529	7-NF2 & 16-NF2	missense_var.	Moderate	M/L
NC_007088.5:6871012	DDB_G0276553	7 & 7-NF2	upstream_gene_var.	Modifier	—
NC_007088.5:7954466	DDB_G0277481	21-NF2	missense_var.	Moderate	M/K
NC_007089.4:1029309	DDB_G0278531	24-NF1	stop_gained	High	K/*
NC_007089.4:1073554	DDB_G0278559	7 & 7-NF2	missense_var.	Moderate	G/S
NC_007089.4:1101637	DDB_G0278575	7 & 7-NF2	upstream_gene_var.	Modifier	—
NC_007089.4:3459101	DDB_G0280505 (*tmem144A*)	5-NF2	missense_var.	Moderate	M/I
NC_007089.4:5126190	DDB_G0281923 (*mrhA*)	21-NF1	downstream_gene_var.	Modifier	—
NC_007089.4:5671190	DDB_G0282355	12 & 12-NF1	upstream_gene_var.	Modifier	—
NC_007090.3:2549009	DDB_G0284845 (*gxcC*)	3-NF1	upstream_gene_var.	Modifier	—
NC_007091.3:905736	DDB_G0287967	18-NF1	downstream_gene_var.	Modifier	—
NC_007091.3:1993104	DDB_G0288805	22	stop_gained	High	K/*
NC_007091.3:2942727	DDB_G0289555 (*arkA*)	21-NF1	missense_var.	Moderate	T/K
NC_007091.3:2996049	DDB_G0289583	11	missense_var.	Moderate	C/Y
NC_007091.3:4205385	DDB_G0290523	21-NF2	synonymous_var.	Low	—
NC_007091.3:4793752	DDB_G0290943 (*pks39*)	20-NF1 & 20-NF3	missense_var.	Moderate	P/S
NC_007091.3:4956884	DDB_G0291085 (*gxcE*)	21-NF2	missense_var.	Moderate	T/I
NC_007092.3:1299121	DDB_G0292198 (RTE)	5-NF2	missense_var.	Moderate	H/L
NC_007092.3:1984519	DDB_G0292696 (*colA*)	9 & 9-NF3	missense_var.	Moderate	P/S

Most SNPs (31 of 38) are in coding regions of the genome, and all but one of these result in an introduced stop codon or amino acid substitution (Table 2). Among the 31 SNPs in coding sequence, 22 are missense variants and according to the Variant Effect Predictor, annotations are predicted to have a moderate impact, 8 others introduce a premature stop codon with a high predicted impact, and the 1 synonymous variant has a low predicted impact. Among the 7 SNPs in noncoding sequence, 5 are upstream and 2 are downstream variants that the Variant Effect Predictor annotated as “modifier,” meaning they are either difficult to predict or that there is no evidence of an impact. This distribution of large effect SNPs strongly supports that they have increased in abundance due to selection, rather than drift.

### Genes with SNPs

The 29 genes with SNPs (Table 2) are distributed throughout the *D. discoideum* genome with between 1 and 8 SNPs on each of the 6 chromosomes. The average GC content for the set of 29 genes (excluding introns) is 28.4% which is close to the genome-wide average of 27% for protein-coding genes. Each of the genes in our list has 1 SNP except for the G protein-coupled receptor (GPCR), *grlG*, which has 10 unique SNPs. GrlG has been proposed to be a candidate folic acid receptor because it is phosphorylated in response to folic acid, but it is not required for eliciting the chemotactic response to folic acid nor has folic acid binding been confirmed, so the function remains uncertain (Pan *et al*. 2016). Among the remaining 28 genes (each with 1 unique SNP), a few have been well described (*e.g. sgmA*, *pks39*, and *arkA*), but the majority (17) are hypothetical proteins still largely lacking annotation.

Using annotations that are available for our 29 genes with SNPs, we carried out functional annotation clustering with DAVID and identified 2 individually significantly enriched annotation clusters. The first cluster of 5 annotations contained UniProt keywords (UniProt Consortium 2023) related to zinc and metal binding (group enrichment score of 1.45) including *arkA*, DDB_G0272484, *rnf160*, DDB_G0269332, and *sgmA*. The second cluster of 10 annotations contained terms related to transmembrane and membrane annotations (group enrichment score of 1.11). This second cluster contained *arkA* and DDB_G0269332 from the first cluster as well as *grlG*, *pks39*, DDB_G0276291, DDB_G0278531, DDB_G0278575, DDB_G0281923, DDB_G0290523, and *tmem144A*. However, after applying the Benjamini correction (Benjamini and Hochberg 1995), none of the individual terms are significantly enriched (Supplementary Table 5). The large number of unannotated genes implicated here hinders interpretation, but it is not unusual given that at the time of writing this manuscript, ∼40% of protein-coding genes in the *D. discoideum* genome still lack annotation.

### SVs provide further support for grlG

We called SVs across all samples (joint calling) using Delly resulting in an unfiltered set of 10,139 SVs. The Delly filter reduced the number to 131, and after all quality filtration, we have a set of 12 SVs (Supplementary Table 6). Among the 12 SVs are 6 deletions, 5 inversions, and 1 duplication. Nine of the 24 evolved lines carry 1 or more of these SVs, 2 of which (lines 1 and 14) did not have any called SNPs. Each SV is unique to 1 evolved line with the exception of 1 deletion (NC_007088.5:1742215–1742506) in *grlG* which was called in lines 17 and 18. However, because we cannot rule out potential contamination of the population sample of line 18, we report only the variant in line 17 (more detail is available in *Variant Read Support* in the Supporting Information). The deletion was verified by PCR in line 17 (as part of the analysis described in the Association of variants with the loss of fruiting body formation section). The SVs range considerably in length (from 183 bp to 49 Kb) potentially impacting as many as 21 different genes. Strikingly, 5 of the 12 SVs impact *grlG*, the same gene that we already identified as carrying 10 SNPs. None of the 28 other genes with SNPs were impacted by any of the SVs. We will only discuss the SVs that impact *grlG* going forward; the full set of 29 SVs that passed filtration are available in VCF format in the Supplementary File 3.

### Some variants in *grlG* are associated with the loss of fruiting body formation

**Fig. 3.**
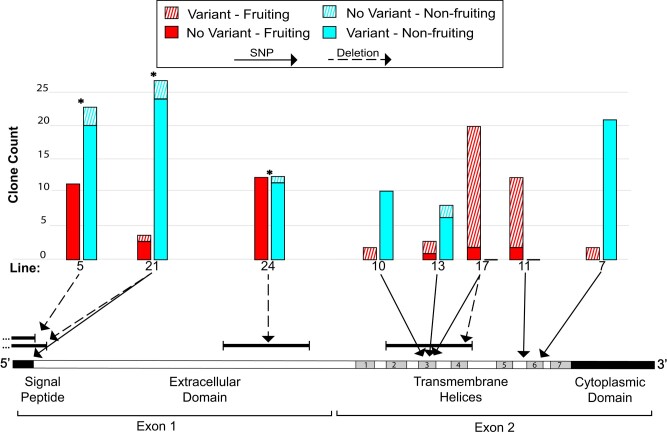
Only variants in the 5′ region of *grlG* are correlated with the loss of cooperative fruiting body formation. Schematic representation (drawn to scale) of the coding sequence of *grlG*. The major features of a mature GrlG protein are indicated below the schematic (see also Fig. 4). Ten variants in 8 different lines (2 lines, 21 and 17, carried both a SNP and a deletion) were included in a genotyping screen of additional clones from each respective evolved line to investigate the association between variants in *grlG* and the clonal ability to form a fruiting body. For each evolved line that we screened clones from, a histogram illustrates the results with side-by-side bars showing the number of clones of the 4 types. A variant is most likely to be causal when most clones either have a variant and do not fruit (solid blue) or do not have variant and do fruit (solid red). Significant associations are indicated by an asterisk (*) above the histogram. Variant locations are indicated with arrows pointing from the histogram to the schematic with either a solid line for SNPs or a dashed line for deletions.

Given the high level of parallelism identified in *grlG* (one or more variants in more than half of the evolved lines), we returned to the evolved lines to screen additional clones for a correlation between variants in *grlG* and the loss of fruiting body formation, as this is one indication of the evolved decrease in cooperation. The variants are located throughout the length of the gene, but a pattern emerged during this screening in which only those variants located in the 5′ half of *grlG* (on exon 1) are associated with the loss of fruiting body formation (*i.e.* non-fruiting clones) and variants in the 3′ half (on exon 2) are not (Fig. 3 and Supplementary Table 2). Because *grlG* has 2 exons of roughly equal length (383 and 390 amino acids for exon 1 and exon 2, respectively) and for simplicity, we will refer to the 2 halves as the 5′ and 3′ regions.

This analysis included 10 of the 15 variants identified across the length of *grlG* that had been called in 8 different lines (2 unique variants were called in lines 21 and 17). In the 5′ region, we screened 88 clones from 3 lines (5, 21, and 24) for the presence of 4 called variants. We found that 88.7% (55 of 62) of the nonfruiter clones carried the variant compared to only 3.7% (1 of 27) of the fruiting clones. The association between the presence of variants in the 5′ region of *grlG* and the clonal phenotype is significant in each evolved line we screened (Fisher's exact *P* < 0.0001, *P* = 0.0164, and *P* < 0.0001 for lines 5, 21, and 24, respectively). In the 3′ region, we screened 78 clones from 5 lines (7, 10, 11, 13, and 17) for the presence of 6 called variants. Unlike the association in the 5′ region, we found the number of clones carrying a variant in the 3′ region was roughly equal between nonfruiters and fruiters [94.8% (37 of 39) and 87.2% (34 of 39), respectively. There is no association between the presence of variants in the 3′ region of *grlG* and the clonal phenotype in any of the evolved lines we screened (Fisher's exact *P* > 0.05).

According to our results, variants in the 5′ region of *grlG* are significantly associated with the non-fruiting phenotype, but not every clone fit that trend (Fig. 3). In all 3 evolved lines that we screened in the 5′ region, we found a few clones that did not have a variant, but they were still unable to form fruiting bodies in isolation. These clones could have lost fruiting body formation due to a different, undetected variant (anywhere in the genome). But clones that are still able to form a fruiting body in isolation, despite the presence of a high impact variant in *grlG*, are more difficult to square with the variant causing non-fruiting (although incomplete penetrance of non-fruiting is a possibility). We found only one such clone in the 5′ region (in line 21), but in the 3′ region, almost all of the fruiting clones that we screened carried a variant in *grlG*. Thus, variants in the 5′ region of *grlG* usually result in the loss of fruiting body formation, but variants in the 3′ region do not.

**Fig. 4.**
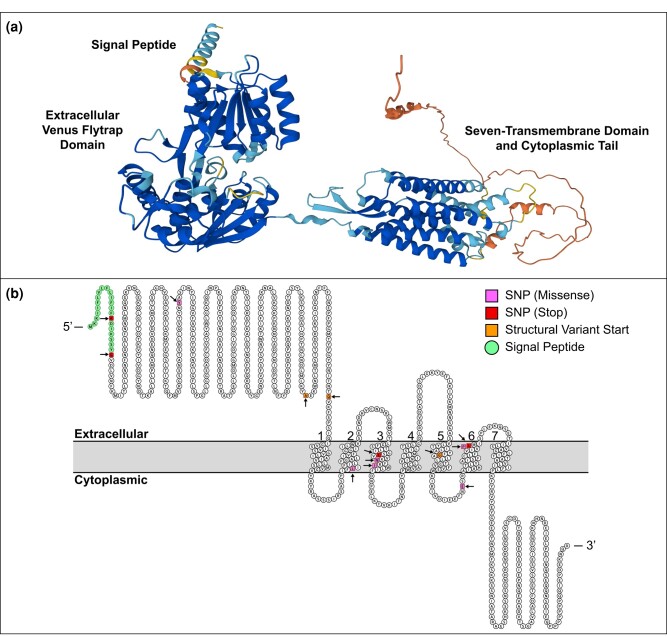
Predicted structure and sequence topology of *D. discoideum* GrlG. a) Predicted folding structure of GrlG generated by AlphaFold (Jumper *et al*. 2021; Varadi *et al*. 2022). Colors indicate the model confidence in the local accuracy; dark blue is very high confidence, light blue is confident, yellow is low confidence, and orange is very low confidence. This structure can be viewed interactively with the 3D viewer on the AlphaFold Protein Structure Database (https://alphafold.ebi.ac.uk/entry/Q75JP4). b) The sequence topology of GrlG generated in Protter (Omasits *et al*. 2014). Extracellular regions are shown above the membrane (in gray) and the cytoplasmic regions below. The helices of the 7-transmembrane domain are numbered 1 through 7. The 5′ amino acids in green indicate the signal peptide. Variant locations are indicated with black arrows, and amino acid colors indicate the type of variant (missense SNPs in purple, stop codons in red, and structural variant start sites in orange; note that 2 structural variants started upstream of the coding sequence and are not depicted here).

To investigate why variants in the 5′ region of *grlG* usually result in the loss of fruiting body formation but variants in the 3′ region do not, we viewed the amino acid sequence and predicted protein structure (Fig. 4). GrlG is one of 17 glutamate receptor-like (“Grl”) proteins (GrlA–H and GrlJ–R) in the *D. discoideum* genome. They are named glutamate receptor-like proteins due to their structural resemblance (and despite little sequence homology) to the glutamate and GABA_B_ receptors in vertebrates and are members of the class C GPCRs (Prabhu and Eichinger 2006; Hall *et al*. 2023). The predicted sequence topology of GrlG shows that it shares the major characteristic features of class C GPCRs including a long 5′ extracellular domain and a 7-transmembrane domain toward the 3′ end followed by an intracellular C-terminal tail (Fig. 4b). And the predicted folding structure generated by AlphaFold (citation) shows the 5′ extracellular region folded with high confidence into a Venus flytrap structure with a clearly visible cleft or ligand binding pocket (Fig. 4a). According to the location of our variants (Fig. 4b), those that impact either the signal peptide or extracellular binding domain are associated with the loss of fruiting body formation, but variants in the 7-transmembrane domain are not. No variants were called in the region encoding the intracellular C-terminal tail.

For a subset of the clones that we screened for *grlG* variants, we also screened for the presence of 1 or more SNPs that were called in other gene(s). These additional screens did not reveal any associations with the clonal phenotype and are therefore only described in the Supporting Information (see also Supplementary Table 2).

### The loss of fruiting body formation is rare in the absence of the social cycle

Non-fruiting clones were rare or absent in the low-relatedness lines that were evolved without the social cycle, suggesting that the social cycle is important. For each of the 3 wild strains (QS6, QS9, and QS18), we clonally plated spores from the ancestor and the 3 replicate evolved lines to estimate the prevalence of non-fruiting clones. Across hundreds of clones screened, we did not observe a single clone which could not form fruiting bodies at all. However, a minority of evolved clones exhibited aberrant fruiting body morphology but always with some identifiable stalks and sori. Even these clones were rare—fewer than 1% of the total clones screened from QS6 and QS9 (*n* = 958, 324 clones) and 7.5% of clones from QS18 (*n* = 778 clones) formed aberrant fruiting bodies.

Even under the most liberal interpretation in which clones that produced aberrant fruiting bodies are considered nonfruiters, these results stand in contrast to the results of Kuzdzal-Fick *et al*. (2011). We found aberrant fruiting body formation in an average of 3.1% of all clones screened from these lines, while clones with a total loss of fruiting body formation accounted for an average of 31% of all clones from the evolved lines of Kuzdzal-Fick *et al*. (2011). Thus, the loss of fruiting body formation did not evolve in the absence of any selective advantage to cheat (by undercontributing to stalk) during the social cycle.

### The genes discovered in this study have not previously been implicated in conflict or cheating during multicellular development

We next turned to the literature to see if any of the 29 genes with variants have been previously implicated in conflict during multicellular development in *D. discoideum*. Most notably missing from our list is *fbxA*, the only gene known to be associated with both cheating and the loss of fruiting body formation (Ennis *et al*. 2000). It is also interesting that our list does not contain any of the characterized facultative cheater genes such as *chtB*, *chtC*, *dimA*, or *csA* (Queller *et al*. 2003; Foster *et al*. 2004; Khare and Shaulsky 2010; Santorelli *et al*. 2013). Aside from that handful of characterized genes, most genes with potential involvement in cheating and conflict in *D. discoideum* have not yet received attention at the individual level. To also look for our genes among that uncharacterized majority, we compiled 4 sets of genes from the literature that have either had mutants directly implicated in cheating (Santorelli *et al*. 2008) or that we deemed likely to contain genes involved in conflict or cheating because of increased expression in chimeras (Hirose *et al*. 2015; Noh *et al*. 2018; de Oliveira *et al*. 2019). Altogether, the list is composed of 591 unique genes (Supplementary Table 7). None of the 29 genes identified in this study were found on that list, and there is little overlap among the 4 gene sets (Supplementary Fig. 1) (additional details are available in the Supporting Information). While surprising, this is in line with previous reports of limited overlap among sets of social genes (kin discrimination, cooperation, and cheating) (de Oliveira *et al*. 2019 ; Noh *et al*. 2020). The limited overlap among these genes further highlights the polygenic and complex nature of multicellular development.

## Discussion

In this study we identified SNPs in 29 genes that arose across 24 experimentally evolved lines of *D. discoideum* from Kuzdzal-Fick *et al*. (2011). Over the course of about 290 generations, lines evolved under conditions of low relatedness became less cooperative (cheated more in mixtures and fruited less well alone). Because relatedness was low, these non-fruiting clones were able to persist only by forming chimeric fruiting bodies with others lacking the same mutations.

Here, we used whole-genome sequencing and variant analyses of those previously evolved cell lines to identify which genes had changed and might be responsible for the observed decrease in cooperation. We identified at least 1 SNP in 17 of the 24 experimentally evolved lines for a total of 38 SNPs. Most of the SNPs (31) are in coding regions of the genome, and all but one results in either an introduced stop codon or other amino acid substitutions, suggesting selective pressure acting on these loci. The majority of the genes with SNPs are not well characterized, and many lack any annotation, which impacted our ability to assess similarities among them.

While 28 of the genes we identified included only a single SNP each, 1 gene—*grlG*—was found to contain 10 unique SNPs across multiple lines. The *grlG* gene also stood out because it harbors 5 structural variants, whereas none of the other genes with SNPs had any. Our results provide strong evidence of parallel evolution of *grlG* and suggest the involvement of this GPCR in cooperation and multicellular development in *D. discoideum*.

### Confidence in variant calls and detection

The *D. discoideum* genome has features that may have impacted our ability to detect variants. The AT content of the *D. discoideum* genome is very high, >77% (Eichinger *et al*. 2005). There is also a high density of simple sequence repeats (DNA tracts of 1–6 bp tandemly repeated a varying number of times) (Tian *et al*. 2011) which represent 14.3% of the genome (Srivastava *et al*. 2019), including in over 16.3% of protein-coding genes (Eichinger *et al*. 2005). The accuracy of variant calling is decreased in low-complexity and repeat regions. We dealt with the challenging features of the *D. discoideum* genome by carefully exploring the effects of different settings for variant calling and filtering to determine a stringent and robust pipeline (see Materials and methods). Despite the challenging features of the *D. discoideum* genome, we are confident that we obtained a high-quality and strongly supported set of variants.

Our stringent pipeline decreased the likelihood of obtaining false positives and ensured that variants called at the population level tend to be those most strongly selected. The tradeoff of such a stringent approach is a decreased power to detect variants, especially low-frequency variants in the population samples. We might miss some variants that arose later in the experiment or those not facing strong selection (the latter being less interesting). We did detect some low-frequency variants by sequencing individual non-fruiting clones from the lines. However, the fact that not every nonfruiter had a called variant shows that we did not detect every non-fruiting mutation.

### Cooperation, non-fruiting, and cheating

The work of Kuzdzal-Fick *et al*. (2011) showed that experimental evolution under low relatedness profoundly decreased cooperation, as evidenced by the widespread loss of fruiting body formation in many clones and the increased prevalence of cheating in many of the lines. Given that nonfruiter clones were incapable of producing fruiting bodies within clonal aggregates, their prevalence within these lines must reflect success achieved within mixtures, possibly by cheating. This is supported by the observation from Kuzdzal-Fick *et al*. (2011) that the proportion of non-fruiting clones tended to be lower or absent in lines that did not cheat (Fig. 2). The original study also tested a small number of non-fruiting clones for the ability to cheat their ancestors and concluded that 75% were not only capable of cheating but that as their proportion within mixtures increased, total spore production declined.

While the loss of fruiting body formation is not necessarily an indication of cheating, selection for cheating is the most likely explanation for the high prevalence of non-fruiting clones. The low-relatedness conditions of the experiment meant that any such mutants were thoroughly mixed with the rest of the population. Therefore, they did not experience the cost of not being able to fruit alone and did experience a steady supply of other clones to exploit.

To further test the hypothesis that the prevalence of nonfruiters in the low-relatedness treatment was the result of selection for cheating, we also screened lines that were experimentally evolved under low-relatedness conditions similar to those used in Kuzdzal-Fick *et al*. (2011), but where passages were performed every 48 hours, before the *D. discoideum* enter the social stage of the life cycle and produce multicellular fruiting bodies. Under these conditions, there would be no selective advantage for mutations which caused cheating, and accordingly, we found that nonfruiters did not arise as they had in Kuzdzal-Fick *et al*. (2011). Given that the key difference between these 2 experimental evolution experiments was the presence or lack of the multicellular development stage, we take these results as support that non-fruiting clones throve in Kuzdzal-Fick *et al*. (2011) due to the advantages they gained from cheating other clones within chimeric fruiting bodies.

### Parallel evolution of grlG

The concentration of highly supported and unique variants in *grlG* across 14 of the 24 replicate lines is strong evidence of parallel evolution at the gene level (Tenaillon *et al*. 2012; Barrick and Lenski 2013; Van den Bergh *et al*. 2018). None of these mutations were present in the ancestral genome, indicating that they occurred during the course of experimental evolution. At the nucleotide level, there is no indication of a mutational hotspot because the variants are located throughout the length of the gene (1,599 bp of coding sequence). Moreover, the variants in *grlG* are composed of several types of mutations including 10 nonsynonymous SNPs that result in either an introduced stop codon (4) or a missense mutation (6), as well as 4 deletions and 1 inversion, all of which impact coding sequence. The number of large effect mutations (half of which result in protein truncation) and the complete lack of synonymous mutations in *grlG* suggests that the loss of *grlG* was adaptive under the experimental conditions. Further support of selection acting on *grlG* is the high population level frequency that several of these new mutations reached in their respective lines. On average, the 10 unique SNPs in *grlG* are supported by 61% of the total reads in their respective populations compared with an average of only 41% support for the 28 remaining SNPs in other genes.

By screening additional clones from the experimental lines, we found that variants in the 5′ region of *grlG* encoding the signal peptide and extracellular binding domain are significantly associated with the loss of fruiting body formation, while those in the 3′ region encoding the 7-transmembrane domain are not. Most of the clones screened in the 3′ region (both fruiting and non-fruiting) carried a variant in *grlG* (91%), which may have limited our power to detect an association. We were unable to find any non-fruiting clones for evolved lines 11 and 17, but the large proportion of normally fruiting clones that carry a *grlG* variant certainly suggests a lack of an association in those lines. Moreover, the variants in both regions of *grlG* were positively selected and increased in abundance under the experimental conditions.

Based on sequence homology and the predicted folding structure (Fig. 4), GrlG appears to function like other class C GPCRs. Typically, ligand binding occurs via the Venus flytrap domain (similar to the ancestral periplasmic binding proteins of bacteria) situated in the 5′ extracellular domain (Cao *et al*. 2009; Chun *et al*. 2012). Upon binding its ligand, conformational changes throughout the 7-transmembrane domain lead to the activation of G proteins (or other effectors) at the intracellular C-terminus to induce signaling inside the cell (Bockaert and Pin 1999; Rosenbaum *et al*. 2009). It is not surprising that the loss of the binding domain, such as in our 5′ variants, would render a receptor nonfunctional. This has been experimentally demonstrated for the homologous protein, GrlL (Far1) (Pan *et al*. 2016). But while we might predict that variants in the transmembrane domain, particularly those also resulting in early protein truncation, would lead to similar outcomes, our data do not support this.

GPCR signaling is known to be complex and pleiotropic in nature, which can impose challenges for predicting variant impacts. Mutations in membrane proteins can result in a huge variety of impacts, far beyond simple gain or loss of function. Not only will the impact depend on the protein properties and the type and location of the mutation, but it may be affected by interacting ligand(s), effector(s), or other proteins (Schöneberg and Liebscher 2021; Zaucha *et al*. 2021). Some of the glutamate receptor-like proteins in *D. discoideum* exemplify the complexity of GPCR signaling. There are examples of glutamate receptor-like proteins that recognize more than one ligand (Anjard and Loomis 2006; Pan *et al*. 2016, 2018) or couple with more than one G protein (Anjard and Loomis 2006; Anjard *et al*. 2009), and there are ligands that can bind to multiple receptors (Wu and Janetopoulos 2013). In addition, there are examples of suspected redundancies, wherein the loss of the focal receptor does not completely abolish the response (Robery *et al*. 2013; Pan *et al*. 2018; Tang *et al*. 2018).

The parallel evolution of *grlG* suggests that the loss of *grlG* was adaptive under the experimental conditions of low relatedness. And although variants in both the 5′ and 3′ regions of *grlG* were positively selected and increased in abundance, it is unclear why only the variants in the 5′ region are associated with the loss of fruiting body formation. Given the complexity of GPCR signaling and the many unknowns, it is difficult to speculate, but perhaps all variants in *grlG* provided the selective advantage of cheating, but only those that inhibit ligand binding are also likely to result in non-fruiting when in isolation. Further work will be needed to resolve this question.

### The potential roles of GrlG and the GPCRs in multicellular development

GPCRs like *grlG* are the largest class of receptors of extracellular stimuli in eukaryotes. The *D. discoideum* genome has a surprisingly large and diverse repertoire of more than 55 GPCRs with representative members from 5 of the 6 major classes (Eichinger *et al*. 2005; Prabhu and Eichinger 2006; Hall *et al*. 2023). They are involved in a diversity of biological processes, but interestingly, the expression profiles suggest that the majority may be involved in multicellular development (Hall *et al*. 2023). Most notably, the class E GPCRs are the receptors of cAMP, which is pivotal in the initiation and coordination of multicellular development in *D. discoideum* (Saran *et al*. 2002; Loomis 2014). GrlG is one of the 17 glutamate receptor-like proteins (GrlA–H and GrlJ–R) in *D. discoideum*, members of the class C GPCRs (Eichinger *et al*. 2005; Prabhu and Eichinger 2006). For most of the group, the ligands, effectors, and even the specific signaling pathways remain uncertain. However, at least 4 have been described as having roles in development including GrlA (Prabhu *et al*. 2007a; Anjard *et al*. 2009), GrlB (Wu and Janetopoulos 2013), GrlE (Anjard and Loomis 2006; Wu and Janetopoulos 2013), and GrlJ (Prabhu *et al*. 2007b). Thus, while much remains to be learned, it is easy to conceive that GrlG is also involved in cooperation and multicellular development.

We are aware of only 1 previous study that directly investigated the function of GrlG, and it was in the context of predation rather than development. Upon exposure to folic acid (a chemoattractant used by *D. discoideum* to chemotax toward and phagocytose bacteria), Pan *et al*. (2016) identified increased phosphorylation at potentially key serine residues in both GrlL and GrlG and both were thus investigated as candidate folic acid receptors (“Far”). However, only GrlL (Far1), and not GrlG (Far2), was required for eliciting the chemotactic response to folic acid and was confirmed to bind folic acid (Pan *et al*. 2016, 2018). The loss of GrlL (Far1) did not completely abolish the response to folic acid, which suggests that other proteins are involved (Pan *et al*. 2018). That is in line with some earlier studies that also suggested that there are 2 folic acid receptors, each with a different binding affinity that are likely utilized at different stages (de Wit and van Haastert 1985; Segall *et al*. 1988).

The other limited data that might support *grlG* as a folic acid receptor can be challenging to interpret. This is especially true for gene expression data, which can show great variability between experiments, and even between replicates (Hall *et al*. 2023). Some experiments have shown that *grlG* peaks in expression during the vegetative stage (Rosengarten *et al*. 2015; Katoh-Kurasawa *et al*. 2021), tracking expectations for a gene involved in sensing and phagocytosing prey. However, other studies have shown that peak expression occurs 12 h after starvation during the tight aggregate stage (Prabhu *et al*. 2007b; Hirose *et al*. 2015), which is the stage at which our non-fruiting clones halt development. A recent RNA-seq study reported that several glutamate receptor-like genes including *grlL* (*far1*) and *grlG* are down-regulated upon exposure to one or more bacterial species or folic acid and suggest that this is new data supporting them both as folic acid receptors (Lamrabet *et al*. 2020). However, the response of *grlL* (*far1*) to the folic acid treatment was not significant and the main RNA-seq results for *grlG* could not be validated by qRT-PCR.

We have no other reason to believe that the parallel evolution of *grlG* that we have identified is related to the detection or phagocytosis of bacterial prey, but we do not dismiss the possibility. The *D. discoideum* AX4 lines were adapted to the laboratory growth and nutrient conditions well before the start of experimental evolution by Kuzdzal-Fick *et al*. (2011). Instead, the primary novelty of the experimental environment was the extremely low relatedness that favored cheaters. Given our understanding of the complex nature of GPCR signaling, even if GrlG was confirmed to be involved in the response to bacterial prey, that would not contradict the evidence we present here suggesting its involvement in cooperation and multicellular development. Instead, perhaps the knowledge that GrlG and other members of the glutamate receptor-like protein family sometimes show low levels of overlapping functionality may help explain why the loss of *grlG* in our evolved clones did not always lead to a total loss of fruiting body formation.

## Conclusion

Kuzdzal-Fick *et al*. (2011) demonstrated how low relatedness in a natural population can lead to a collapse of multicellularity and, with it, the advantages of fruiting body formation and spore dispersal. We used whole-genome sequencing of those cell lines to identify genetic variants that had increased and identified widespread parallel evolution of *grlG* encoding an orphan GPCR. By screening and genotyping an additional 167 clones from the evolved lines, we identified a significant correlation between the loss of fruiting body formation and the presence of variants in the 5′ region of *grlG* encoding the signal peptide and extracellular binding domain. Two puzzles in our results warrant further research. First, though variants in both the 5′ and 3′ regions of the gene increased in frequency, why did only the variants in the 5′ show a correlation with the non-fruiting phenotype? Second, how do these results square with prior suggestions that GrlG might be involved in the very different function of folate sensing of bacterial prey? However, this work brings us one step closer to deorphanization of another of the ever-important GPCRs and highlights the need to expand research efforts to characterize the remaining GPCRs in *D. discoideum*.

## Data Availability

Sequence data are publicly available under the GenBank BioProject PRJNA996142 including the reads in BAM format aligned to the concatenated *D. discoideum* and *K. pneumoniae* reference genomes on the Sequence Read Archive (SRA). All cell lines used in this study are in the lab of the senior authors (J.E.S. and D.C.Q.) and are available upon request. Supplementary Materials are available through the GSA figshare: https://gsajournals.figshare.com/articles/journal_contribution/Supplemental_Material_for_Walker_et_al_2023/23749284. Supplementary File 1 contains all Supporting Information and Supplementary Figs. 1–3. Supplementary File 2 contains all Supplementary Tables 1–7 on separate tabs (in .xlsx format). Supplementary File 3 is a compressed folder that contains 3 VCF files, each containing all variant calls that survived hard filtration (before manual review) from each of the 3 variant callers (GATK, Freebayes, and Delly). Supplementary File 4 contains the *D. discoideum* gene annotations (in .txt format) downloaded from NCBI on 2019 October 25. Supplemental material available at G3 online.
